# MRPL23 Overexpression Predicts Poor Survival and Is Associated with Mitochondrial Respiratory Signatures in Glioblastoma

**DOI:** 10.3390/cancers18142226

**Published:** 2026-07-10

**Authors:** Justyna Durślewicz, Marek Zdrenka, Łukasz Szylberg, Jędrzej Borowczak

**Affiliations:** 1 Faculty of Medicine, Bydgoszcz University of Science and Technology, Aleje Prof. S. Kaliskiego 7, 85-796 Bydgoszcz, Poland; jedrzej.borowczak@pbs.edu.pl; 2 Department of Tumor Pathology and Pathomorphology, Oncology Centre—Prof. Franciszek Łukaszczyk Memorial Hospital, 85-796 Bydgoszcz, Poland; zdrenkam@co.bydgoszcz.pl (M.Z.); l.szylberg@cm.umk.pl (Ł.S.); 3 Department of Obstetrics, Gynaecology and Oncology, Ludwik Rydygier Collegium Medicum in Bydgoszcz, Nicolaus Copernicus University in Toruń, 85-168 Bydgoszcz, Poland; 4 Clinical Department of Oncology, Oncology Centre—Prof. Franciszek Łukaszczyk Memorial Hospital, 85-796 Bydgoszcz, Poland

**Keywords:** glioblastoma, MRPL23, prognostic biomarker

## Abstract

Background: Glioblastoma (GBM) is the most aggressive primary brain tumor in adults and is characterized by poor prognosis and marked molecular heterogeneity. Mitochondrial ribosomal proteins have emerged as regulators of cancer metabolism, yet the clinical significance of MRPL23 in GBM remains unclear. This study aimed to evaluate the prognostic relevance of MRPL23 expression in glioblastoma and its association with patient survival and mitochondrial functional profiles. Methods: MRPL23 protein expression was assessed by immunohistochemistry in tissue microarrays derived from 89 patients with primary glioblastoma and 36 samples of adjacent non-tumorous brain tissue. Survival analyses were performed using Kaplan–Meier estimates and Cox proportional hazards models. In silico validation was conducted using transcriptomic and proteomic data from 296 IDH-wildtype glioblastomas from The Cancer Genome Atlas. Correlation analyses were used to explore associations between MRPL23 expression and mitochondrial respiratory chain components. Results: MRPL23 protein expression was significantly upregulated in glioblastoma compared with non-tumorous brain tissue (*p* < 0.001). High MRPL23 expression was associated with significantly shorter overall survival in the institutional cohort (median 13.5 vs. 21 months, *p* = 0.013). Consistently, low MRPL23 expression in the TCGA cohort was associated with improved overall and progression-free survival. MRPL23 expression showed strong positive correlations with key mitochondrial respiratory chain proteins, including COX5B, UQCRC1, and COX4I1. Conclusions: MRPL23 is overexpressed in glioblastoma and is associated with unfavorable patient outcomes. These findings identify MRPL23 as a potential prognostic biomarker and highlight mitochondrial translation as a relevant biological process in aggressive glioblastoma.

## Simple Summary

Glioblastoma is the most aggressive brain cancer in adults and remains difficult to treat despite advances in surgery, radiotherapy, and chemotherapy. Identifying biomarkers associated with patient survival may improve prognostic assessment and support the development of new treatment strategies. In this study, we investigated the mitochondrial ribosomal protein MRPL23 in glioblastoma tissues and public genomic datasets. We found that MRPL23 was expressed at higher levels in glioblastoma than in normal brain tissue and that elevated expression was associated with shorter patient survival. Furthermore, MRPL23 expression correlated with proteins involved in mitochondrial energy production, suggesting a role in tumor metabolism. These findings indicate that MRPL23 may serve as a novel prognostic biomarker and highlight mitochondrial translation as a potentially important therapeutic target in glioblastoma.

## 1. Introduction

Glioblastoma (GBM) is the most common and the most aggressive primary brain tumor in adults, accounting for nearly 50% of malignant gliomas [1,2]. Despite standard multimodal treatment, including maximal safe resection, radiotherapy, and temozolomide chemotherapy, the prognosis of GBM patients remains extremely poor. The median overall survival (OS) is approximately 15 months, and the 5-year survival rate remains below 10% [3,4]. This unfavorable outcome is largely attributed to the remarkable molecular heterogeneity, highly infiltrative growth, and intrinsic therapeutic resistance of GBM cells [5]. Therefore, there is an urgent need to identify novel prognostic biomarkers and therapeutic targets to improve patient stratification and clinical management.

Mitochondria are central organelles regulating oxidative metabolism, redox balance, apoptosis, and nucleotide biosynthesis. In cancer, mitochondrial dysfunction and altered mitochondrial translation contribute to metabolic reprogramming and tumor progression [6]. Mitochondrial ribosomal proteins (MRPs), which form the structural backbone of mitochondrial ribosomes, are indispensable for the synthesis of proteins encoded by the mitochondrial genome. Because mitochondrially encoded proteins constitute essential subunits of the oxidative phosphorylation system, dysregulated MRP expression may alter respiratory chain activity, ATP production, redox homeostasis, apoptosis regulation, and metabolic plasticity. Through these mechanisms, aberrant expression of MRPs may support tumor cell proliferation, survival under stress conditions, apoptosis resistance, invasive behavior, and therapeutic resistance. Recent studies indicate that MRPs are dysregulated in multiple cancer types and may therefore serve as potential prognostic biomarkers and therapeutic targets in oncology [6,7,8]. MRPL23, a mitochondrial ribosomal protein of the large subunit, plays an essential role in mitochondrial translation and mitochondrial homeostasis [9]. Its deregulation has been implicated in several malignancies. For example, overexpression of MRPL23 correlates with poor survival in clear cell renal cell carcinoma [7], and experimental studies show that MRPL23 depletion induces senescence and loss of proliferative capacity [8]. Moreover, integrative omics analyses suggest that mitochondrial ribosomal proteins are linked to prognostic signatures and therapeutic vulnerabilities in glioblastoma [5]. However, the clinical significance of MRPL23 in GBM remains largely unexplored, particularly at the protein level and in relation to patient outcomes.

In this study, we investigated the prognostic role of MRPL23 expression in GBM using in-house and publicly available datasets. We hypothesized that MRPL23 may serve as a novel prognostic biomarker, and aimed to analyze its association with clinicopathological features, overall survival, and potential functional interactions related to mitochondrial function in GBM.

## 2. Materials and Methods

### 2.1. Patients and Tissue Specimens

Formalin-fixed paraffin-embedded (FFPE) tumor tissue specimens were obtained from 89 patients with histologically confirmed GBM, who underwent surgical resection at the 10th Military Research Hospital and Polyclinic in Bydgoszcz, Poland, between 2019 and 2020. The control group consisted of 36 samples of adjacent non-tumorous brain tissue collected from the same patients.

Tissue macroarrays (TMAs) were constructed from formalin-fixed paraffin-embedded donor blocks. Representative tumor areas were selected on hematoxylin and eosin-stained sections by an experienced pathologist and then matched to the corresponding regions in the donor paraffin blocks. One representative tissue core was obtained from each case and transferred into a recipient paraffin block. Clinical data included age (median 59 years, range 27–90 years), sex, MGMT promoter methylation, 1p/19q codeletion, tumor localization, proximity to the ventricular system, and extent of resection. All tumors were reclassified according to the 5th edition of the WHO Classification of Tumors of the Central Nervous System (2021) [1]. The study was conducted in accordance with the Declaration of Helsinki and was approved by the local Institutional Ethics Committee.

### 2.2. Immunohistochemistry

For immunohistochemical analysis, 4 μm sections were prepared from representative FFPE tumor blocks using an Accu-Cut rotary microtome (Sakura Finetek USA, Inc., Torrance, CA, USA). Staining was performed on the BenchMark^®^ ULTRA PLUS automated platform (Roche Diagnostics/Ventana Medical Systems, Tucson, CA, USA). MRPL23 was detected with a rabbit polyclonal anti-MRPL23 antibody (cat. no. HPA050406, Sigma-Aldrich, St. Louis, MO, USA), used at a 1:100 dilution and incubated for 32 min at room temperature. Signal development was performed with the ultraView Universal DAB Detection Kit (Roche Diagnostics/Ventana Medical Systems, Tucson, AZ, USA), following the manufacturer’s recommendations. Finally, the slides were counterstained with hematoxylin, dehydrated, and coverslipped. Digitized slides were reviewed using scans obtained with the Ventana DP 600 slide scanner (Roche Diagnostics/Ventana Medical Systems, Tucson, AZ, USA). MRPL23 staining was assessed independently by a board-certified pathologist (M.Z.) and an experienced researcher (J.D.), both blinded to clinical information. Protein expression was evaluated semi-quantitatively using an immunoreactive scoring approach that combined staining intensity with the proportion of positive tumor cells. Staining intensity was assigned a score from 0 to 3, corresponding to absent, weak, moderate, or strong staining, respectively. The extent of immunoreactivity was scored from 0 to 4 according to the percentage of positive cells: 0 for no positive cells, 1 for ≤10%, 2 for 11–50%, 3 for 51–80%, and 4 for >80%. The final immunoreactive score was obtained by multiplying the intensity score by the extent score, resulting in values ranging from 0 to 12. For subsequent analyses, cases were categorized as low or high MRPL23 expression using the median score as the cutoff.

### 2.3. In Silico Analysis

Clinical data from The Cancer Genome Atlas (TCGA) Glioblastoma (GBM) cohort were retrieved via the cBioPortal platform [10]. Transcriptomic and protein-level data were obtained from the University of California, Santa Cruz (UCSC) Xena browser (University of California, Santa Cruz, CA, USA; GDC TCGA Glioblastoma [GBM] datase) [11]. MRPL23 mRNA expression was extracted from the STAR-FPKM RNA-seq dataset available through the GDC Hub (National Cancer Institute, Bethesda, MD, USA), whereas protein expression data for selected mitochondrial respiratory chain-related proteins were obtained from the Protein Expression Quantification dataset available thought the same platform. Clinical, transcriptional, and protein-level data were matched using TCGA patient/sample barcodes. Of the initial 517 TCGA-GBM cases, IDH-mutant cases were excluded to approximate the 2021 WHO definition of adult-type glioblastoma, and cases with insufficient clinical or survival data were removed. The final dataset included 296 cases [1]. Because the availability of RNA-seq, protein expression, survival, and treatment data differed across cases, analyses were performed using complete cases for each specific endpoint. For survival analysis and stratification, MRPL23 expression was dichotomized using the Cutoff Finder online tool (Charité–Universitätsmedizin Berlin, Berlin, Germany), and the cutoff was set at FPKM = 2727 [12]. Correlation analyses were performed between MRPL23 mRNA expression and protein expression of selected mitochondrial respiratory chain-related proteins in cases with available paired transcriptomic and protein-level data.

### 2.4. Statistical Analysis

Statistical analyses were performed using GraphPad Prism version 9.0 (GraphPad Software, San Diego, CA, USA) and Statistica version 13.3 (TIBCO Software Inc., Palo Alto, CA, USA). The distribution of continuous variables was evaluated with the Shapiro–Wilk test. Because most variables did not follow a normal distribution, group comparisons for continuous data were performed using the Mann–Whitney U test. Associations between categorical variables were assessed using the chi-square test or Fisher’s exact test, as appropriate. Overall survival was analyzed using the Kaplan–Meier method, and differences between survival curves were compared with the log-rank test. Cox proportional hazards regression models were applied to estimate hazard ratios (HRs) with corresponding 95% confidence intervals (CIs) in univariate and multivariate analyses. A *p*-value below 0.05 was considered statistically significant. For the TCGA-GBM cohort, variables associated with overall survival in univariate Cox regression analysis at *p* < 0.10 were included in the multivariate Cox proportional hazard model. Age was analyzed as a continuous variable, while radiotherapy status was coded as no versus yes and MRPL23 mRNA expression as high versus low. Variables with *p* ≥ 0.10 in univariate analysis were not included in the multivariate model.

## 3. Results

### 3.1. MRPL23 Protein Expression in GBM and Adjacent Non-Tumorous Brain Tissue

**Figure 1:**
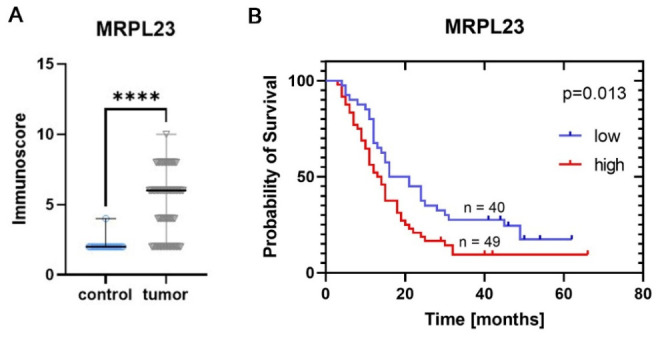
MRPL23 protein expression and its prognostic relevance in the TMA cohort. (**A**) MRPL23 protein expression is significantly upregulated in glioblastoma compared with adjacent non-tumorous brain tissue. (Quantitative analysis of immunoreactive score, IRS.) (**B**) Kaplan–Meier analysis of overall survival according to MRPL23 protein expression in the TMA cohort. (MRPL23-low vs. MRPL23-high.) **** *p* < 0.0001.

**Figure 2:**
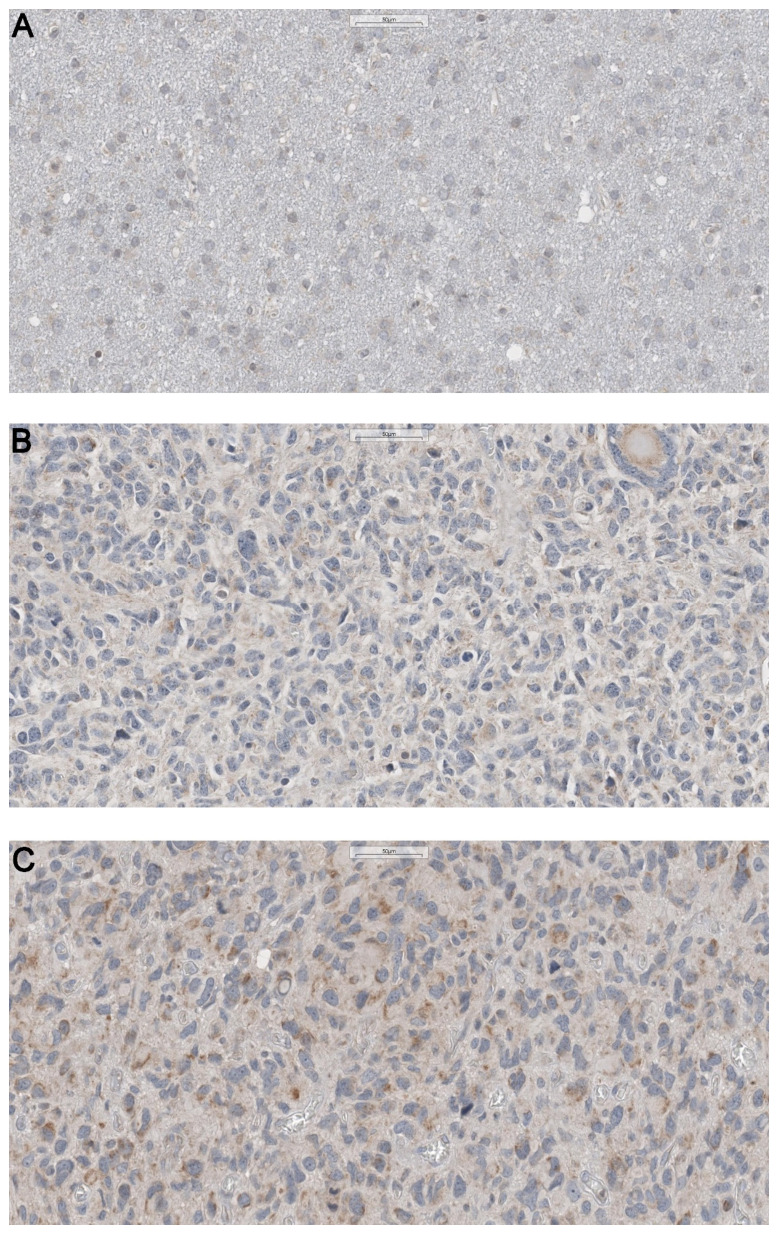
Representative immunohistochemical staining of MRPL23 in glioblastoma and adjacent non-tumorous brain tissue. (**A**) Adjacent non-tumorous brain tissue; (**B**) glioblastoma with low MRPL23 expression; (**C**) glioblastoma with high MRPL23 expression.

IHC staining demonstrated that MRPL23 was predominantly localized to the cytoplasm of GBM cells. Quantitative analysis revealed a significant upregulation of MRPL23 protein expression in GBM tissues compared with adjacent non-tumorous brain tissues (*p* < 0.001; Figure 1A). Adjacent non-tumorous brain tissue consistently exhibited weak cytoplasmic immunoreactivity, whereas GBM tissues showed markedly elevated cytoplasmic expression (Figure 2).

### 3.2. Association Between MRPL23 Expression and Clinicopathological Characteristics in the TMA Cohort

The analysis included 89 patients with primary glioblastoma who underwent complete resection (Table 1). Of these, 41 exhibited low MRPL23 protein expression (IRS ≤ 4) and 48 showed high MRPL23 protein expression (IRS > 4). No statistically significant correlations were observed between MRPL23 protein expression and clinicopathological features, including age, sex, tumor location, performance status, smoking history, or antiepileptic drug use (all *p* > 0.05). The only significant association was observed with MGMT promoter methylation status, with higher MRPL23 protein expression detected in tumors with intermediate MGMT methylation compared with MGMT-unmethylated samples (*p* = 0.02).

### 3.3. Prognostic Significance of MRPL23 Expression in the TMA Cohort

Kaplan–Meier survival analysis demonstrated a significantly shorter overall survival in patients with high MRPL23 protein expression compared with those with low expression (median OS: 13.5 vs. 21 months; log-rank *p* = 0.013; Figure 1B). In univariate Cox proportional hazards regression analysis, increasing age (HR = 1.03 per year; 95% CI: 1.01–1.05; *p* = 0.006) and high MRPL23 protein expression (HR = 1.76; 95% CI: 1.22–2.83; *p* = 0.016) were associated with shorter overall survival in glioblastoma patients. MGMT promoter methylation status showed a significant protective effect for tumors with intermediate methylation compared with MGMT-unmethylated cases (HR = 0.38; 95% CI: 0.23–0.62; *p* = 0.001), whereas low MGMT methylation was not associated with survival (*p* = 0.44). Sex, tumor hemisphere, Karnofsky Performance Status, smoking status, and antiepileptic drug use were not significantly associated with overall survival (Table 2).

In multivariate Cox regression analysis, intermediate MGMT promoter methylation (HR = 0.48; 95% CI: 0.28–0.82; *p* = 0.004) and age (HR = 1.02; 95% CI: 1.01–1.05; *p* = 0.037) remained an independent prognostic factors. The association between high MRPL23 expression and overall survival lost statistical significance after adjustment for confounding variables (HR = 1.54; 95% CI: 0.93–2.56; *p* = 0.10).

### 3.4. In Silico Validation of MRPL23 mRNA Expression in the TCGA GBM Cohort

MRPL23 mRNA expression data were extracted from the TCGA-GBM cohort (Table 3). We found no correlations between MRPL23 mRNA expression, patients’ age, sex, or race (*p* > 0.05). Analyses of the association between MRPL23 mRNA expression and other clinicopathological features of GBM were limited, since the TCGA-GBM dataset does not contain staging information for glioblastoma.

### 3.5. Prognostic Significance of MRPL23 mRNA Expression in the TCGA GBM Cohort

**Figure 3:**
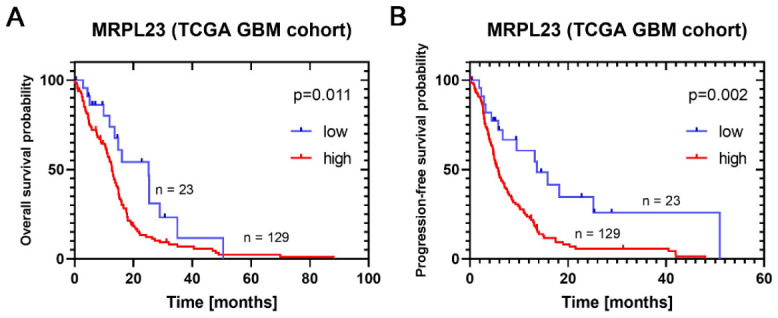
Low MRPL23 expression is associated with longer overall and progression-free survival in the TCGA GBM cohort. (**A**): Overall survival according to MRPL23 expression in the TCGA GBM cohort. (**B**): Progression-free survival according to MRPL23 expression in the TCGA GBM cohort.

Patients with low MRPL23 mRNA expression had significantly higher 2-year overall survival compared to patients with high MRPL23 glioblastomas (54.46% vs. 13.4%; Figure 3A). The 2-year progression-free survival was also higher in MRPL23-low patients (34.44 vs. 6.05%; Figure 3B).

Next, we used Cox proportional hazard regression to explore the prognostic significance of MRPL23 mRNA expression for overall survival in the TCGA-GBM cohort. In univariate Cox regression analysis, age, absence of documented radiotherapy, and high MRPL23 mRNA expression were associated with shorter overall survival, whereas sex was not significant. In the multivariate model including age, radiation status, and MRPL23 mRNA expression, absence of documented radiotherapy and high MRPL23 mRNA expression remained significantly associated with shorter overall survival in the TCGA-GBM cohort (*p* < 0.001, Table 4).

### 3.6. Correlation Profile of MRPL23 mRNA Expression with Mitochondrial Respiratory Chain Protein Levels in the TCGA GBM Cohort

For descriptive interpretation, the strength of correlations was classified according to the absolute value of the correlation coefficient. Correlations were considered weak when |r| was <0.30, moderate when |r| ranged from 0.30 to 0.49, and strong when |r| was ≥0.50. These thresholds were used as interpretative benchmarks and were not intended to imply causality. MRPL23 mRNA expression in the TCGA-GBM cohort showed strong positive correlations with protein expression levels of selected mitochondrial respiratory chain proteins. The highest correlations were observed for COX5B (r = 0.61; 95% CI 0.55–0.66; *p* < 0.0001), UQCRC1 (r = 0.57; 95% CI 0.51–0.63; *p* < 0.0001), and COX4I1 (r = 0.52; 95% CI 0.45–0.58; *p* < 0.0001). A significant positive correlation was also detected with the pro-apoptotic regulator BAX (r = 0.51; 95% CI 0.44–0.57; *p* < 0.0001) and CYCS (r = 0.40; 95% CI 0.32–0.47; *p* < 0.0001). Moderate but significant positive correlations were found with ATP5F1 (r = 0.19; 95% CI 0.11–0.28; *p* < 0.0001) and SDHB (r = 0.26; 95% CI 0.18–0.34; *p* < 0.0001), whereas a weaker yet significant association was observed for NDUFA9 (r = 0.13; 95% CI 0.04–0.22; *p* = 0.002). Interestingly, the only significant negative correlation was identified for NDUFS1 (r = –0.26; 95% CI –0.34 to –0.17; *p* < 0.0001).

## 4. Discussion

This study aimed to elucidate the prognostic and biological role of MRPL23 in glioblastoma. We demonstrated that MRPL23 protein expression is significantly higher in GBM tissues compared with adjacent non-tumorous brain tissue. Importantly, among tumor samples, relatively higher MRPL23 expression was associated with shorter overall survival. These findings suggest that MRPL23 may be linked to a more aggressive GBM phenotype, potentially reflecting enhanced mitochondrial activity and metabolic adaptability.

MRPL23 is a component of the large subunit of the mitochondrial ribosome and supports the synthesis of mitochondrially encoded proteins required for oxidative phosphorylation [9]. Dysregulation of mitochondrial ribosomal proteins has been implicated in cancer-related alterations in bioenergetics, apoptosis resistance, and metabolic homeostasis [6]. Although glioblastoma is commonly associated with glycolytic metabolism, increasing evidence indicates that subsets of glioma cells can retain or reacquire oxidative phosphorylation capacity, particularly under environmental stress conditions [4,13,14,15,16]. Moreover, inhibition of mitochondrial translation suppresses glioblastoma stem-like cell growth, supporting the relevance of mitochondrial function in aggressive GBM phenotypes [17,18,19,20,21,22].

In this context, the association between high MRPL23 expression and poor survival may reflect a metabolically adapted tumor phenotype rather than a direct causal effect. This interpretation is supported by the positive correlations between MRPL23 expression and mitochondrial respiratory chain proteins, including UQCRC1, COX4I1, and COX5B, suggesting preserved respiratory capacity in MRPL23-high tumors. The additional correlations with BAX and CYCS may indicate a link with mitochondrial stress- and apoptosis-related signaling, whereas the negative correlation with NDUFS1 may suggest remodeling of the OXPHOS profile. Collectively, these findings indicate that MRPL23-high GBMs may represent a subgroup characterized by preserved mitochondrial translation, respiratory activity, and metabolic adaptability [20,21,22].

Hypoxia is a central feature of the glioblastoma microenvironment and has been associated with glioma stem-like properties, invasion, angiogenesis, metabolic adaptation, and resistance to radiotherapy and chemotherapy [23,24,25]. Although hypoxia and oxidative phosphorylation are biologically interconnected in glioblastoma progression, to our knowledge, no previous study has directly demonstrated that MRPL23 induces a hypoxic tumor microenvironment or promotes invasion in GBM. Therefore, MRPL23 should not be interpreted as a proven driver of hypoxia or invasion based on the present data. Rather, our findings suggest that MRPL23 may serve as a marker of a metabolically adapted tumor subgroup with preserved mitochondrial translation and respiratory activity. Such a phenotype may facilitate adaptation to microenvironmental and therapeutic stress, including hypoxia-associated stress, but this hypothesis requires validation in functional models. Future studies should assess whether MRPL23 modulation affects hypoxia-related signaling pathways, invasive capacity, and sensitivity to radiotherapy or temozolomide. Taken together, these observations suggest that MRPL23 overexpression should be interpreted primarily as a marker of preserved mitochondrial translation and respiratory activity rather than as direct evidence of hypoxia induction or invasive behavior.

From a mechanistic perspective, maintenance of mitochondrial translation through MRPL23 may provide several advantages to tumor cells. Since mitochondrial translation is required for the synthesis of mitochondrially encoded oxidative phosphorylation subunits, MRPL23-high tumors may have an enhanced ability to preserve mitochondrial bioenergetics and respiratory chain activity [9,17]. This may support improved metabolic adaptability, maintenance of ATP production, regulation of redox balance, preservation of mitochondrial integrity, and generation of biosynthetic intermediates required for nucleotide and lipid synthesis, thereby facilitating rapid tumor growth [21,26,27]. Together, these mechanisms offer a plausible explanation for the more aggressive clinical behavior and worse outcomes observed in patients with MRPL23-high tumors. However, these findings remain correlative, and functional studies are required to determine whether MRPL23 directly promotes glioblastoma progression.

Evidence from non–central nervous system malignancies further supports the biological relevance of MRPL23 and mitochondrial ribosomal protein dysregulation in cancer. In clear-cell renal cell carcinoma, high MRPL23 expression at both the transcript and protein levels has been associated with significantly shorter patient survival and remained prognostically relevant in multivariable analyses [7]. In hepatocellular carcinoma, experimental suppression of MRPL23 induced cellular senescence and reduced proliferative capacity, suggesting that tumor cells may depend on MRPL23 to maintain cellular fitness [8]. These examples indicate that MRPL23 may support tumor progression by sustaining mitochondrial translation, bioenergetic homeostasis, and proliferative capacity. More broadly, dysregulated mitochondrial ribosomal proteins have been linked to altered oxidative metabolism, redox balance, apoptosis resistance, metastatic potential, and therapeutic response across multiple cancer types [6]. In this context, our findings in GBM are consistent with the broader concept that aberrant mitochondrial ribosomal protein expression may provide cancer cells with metabolic and survival advantages.

Of additional interest, MRPL23 is located within the imprinted 11p15.5 chromosomal region, a locus with well-established roles in growth regulation and cancer predisposition. Alterations within this region are implicated in Beckwith–Wiedemann spectrum and Wilms tumor, underscoring the oncogenic relevance of the genomic context in which MRPL23 resides [28,29,30,31]. Although these imprinting abnormalities do not directly establish an oncogenic function for MRPL23, they further support its positioning within a biologically critical regulatory hotspot.

## 5. Conclusions

In conclusion, this study identifies MRPL23 as a potential prognostic biomarker in glioblastoma. A major strength of the study is the combined assessment of MRPL23 protein expression in an institutional GBM cohort and validation using an independent TCGA-GBM dataset. Our findings show that MRPL23 is overexpressed in GBM tissue, is associated with unfavorable survival, and correlates with mitochondrial respiratory chain-related proteins, suggesting a link with metabolic adaptability and preserved mitochondrial respiratory activity.

The study also has limitations, including its retrospective design, limited availability of standardized chemoradiotherapy response data, and the lack of functional validation. Therefore, the observed associations should be interpreted as hypothesis-generating. Future prospective studies should evaluate MRPL23 together with MGMT promoter methylation, treatment-response data, and radiological outcomes. Functional experiments are also needed to determine whether MRPL23 directly affects mitochondrial respiration, invasion, and sensitivity to radiotherapy or temozolomide. If validated, MRPL23 may contribute to improved prognostic stratification and support the development of mitochondria-targeted therapeutic strategies in glioblastoma. 

## Figures and Tables

**Table 1:** Clinicopathological characteristics of glioblastoma patients in the TMA cohort.

Clinical Data		Glioblastoma Cases, *n* (%) *
Cases		89
Mean age (years)		59 (σ ± 12.3)
Sex	Female	38 (42.7%)
	Male	51 (57.3%)
MGMT status	None	10 (11.2%)
	Present, low burden	36 (40.4%)
	Present, medium burden	43 (48.3%)
Brain hemisphere	Left	47 (52.8%)
	Right	43 (47.2%)
Type of procedure	Neuromonitoring	11 (12.3%)
	5-ALA	30 (33.7%)
	Both	16 (18%)
Smoking	No	73 (82%)
	Yes	16 (18%)
Antiepileptic drugs	No	53 (59.6%)
	Yes	36 (40.4%)
Survival status	Alive	14 (15.7%)
	Dead	75 (84.3%)
Overall survival (median)		15 months (IQR 10–28)

* Due to missing data numbers not always add up to 89.

**Table 2:** Univariate and multivariate Cox proportional hazards regression analyses of prognostic factors in the TMA cohort.

Univariate Analysis				Multivariate Analysis		
Predictor	HR	95% CI	*p*-value	HR	95% CI	*p*-value
Age (per year)	1.03	1.01–1.05	0.006	1.02	1.01–1.05	0.037
Sex (Female vs. Male)	0.81	0.51–1.28	0.37	-	-	-
Hemisphere (Left vs. Right)	1.03	0.66–1.63	0.88	-	-	-
MGMT						
Low expression vs. MGMT-unmethylated	0.8	0.39–1.67	0.44	1.04	0.48–2.35	0.74
Medium expression vs. MGMT-unmethylated	0.38	0.23–0.62	0.001	0.48	0.28–0.82	0.004
KPS (linear)	0.99	0.96–1.02	0.38	-	-	-
Smoking (No vs Yes)	1.16	0.65–2.08	0.62	-	-	-
Antiepileptic drugs (No vs. Yes)	1.08	0.67–1.73	0.75	-	-	-
MRPL23 (High vs. low)	1.78	1.11–2.83	0.016	1.54	0.93–2.56	0.1

KPS, Karnofsky Performance Status Scale.

**Table 3:** Clinicopathological characteristics of patients from the TCGA GBM cohort.

Clinical Data		Glioblastoma Cases, *n* (%) *
Cases		296
Median age (years)		62 (IQR 53–72)
Sex	Female	83 (28%)
	Male	118 (39.9%)
Race	White	166 (56.1%)
	Black or African American	24 (8.1%)
	Asian	3 (1%)
Radiotherapy	Yes	156 (52.7%)
	No	27 (9.1%)
Progression	No progression	56 (18.9%)
	Progression	238 (80.4%)
Progression-free survival (median)		5.5 months (IQR 3.06–11.3)
Survival status	Alive	62 (20.9%)
	Dead	232 (78.4%)
Overall survival (median)		11.5 months (IQR 4.77–17.95)

IQR, interquartile range. * The total number of cases does not always sum to 296 due to missing data in the TCGA database and different cases used for protein and mRNA analyses.

**Table 4:** Univariate and multivariate Cox proportional hazards regression analyses for overall survival in the TCGA GBM cohort.

Univariate Analysis				Multivariate Analysis		
Predictor	HR	95% CI	*p*-value	HR	95% CI	*p*-value
Age (per year)	1.04	1.027–1.058	<0.001	1.02	0.997–1.05	0.08
Sex (Female vs. Male)	0.77	0.55–1.08	0.14	-	-	-
RTX (No vs. Yes)	1.76	1.1–2.8	0.017	8.37	2.92–24	<0.001
MRPL23 (High vs. Low)	1.9	1.08–3.32	0.025	9.09	2.97–27.9	0.001

HR, hazard ratio; CI, confidence interval; N/A, not applicable; RTX, radiotherapy.

## Data Availability

Publicly available datasets were analyzed in this study. TCGA-GBM data are available through the UCSC Xena Browser and cBioPortal platforms. Additional data supporting the findings of this study are available from the corresponding author upon reasonable request.
